# Identification of a biomass unaffected pale green mutant gene in Chinese cabbage (*Brassica rapa* L. ssp. *pekinensis*)

**DOI:** 10.1038/s41598-022-11825-1

**Published:** 2022-05-11

**Authors:** Yonghui Zhao, Shengnan Huang, Nan Wang, Yun Zhang, Jie Ren, Ying Zhao, Hui Feng

**Affiliations:** College of Horticulture, Shenyang Agricultural University, Shenyang, 110866 China

**Keywords:** Genetics, Molecular biology, Plant sciences

## Abstract

Chlorophyll (Chl) is an essential component of the photosynthetic apparatus and pigments in plant greening. Leaf color is an important agronomic and commercial trait of Chinese cabbage. In this study, we identified a pale green mutant *pgm* created by ethyl methane sulfonate (EMS) mutagenesis in Chinese cabbage. Compared with wild-type (FT), *pgm* had a lower Chl content with a higher Chl *a*/*b* ratio, imperfect chloroplast structure, and lower non-photochemical quenching. However, its net photosynthetic rate and biomass showed no significant differences. Genetic analysis revealed that the pale green phenotype of *pgm* was controlled by a recessive nuclear gene, designated as *Brpgm*. We applied BSR-Seq, linkage analysis, and whole-genome resequencing to map *Brpgm* and predicted that the target gene was *BraA10g007770.3C* (*BrCAO*), which encodes chlorophyllide a oxygenase (CAO). *Brcao* sequencing results showed that the last nucleotide of its first intron changed from G to A, causing the deletion of the first nucleotide in its second CDS and termination of the protein translation. The expression of *BrCAO* in *pgm* was upregulated, and the enzyme activity of CAO in *pgm* was significantly decreased. These results provide an approach to explore the function of *BrCAO* and create a pale green variation in Chinese cabbage.

## Introduction

Chlorophyll (Chl) is a green pigment in higher plants and other photosynthetic organisms and plays an essential role in photosynthesis by capturing light energy and converting it into chemical energy^1^. There are two kinds of Chl in higher plants, Chl *a* and Chl *b*. Chl *a* and Chl *b* have the same chemical structure except in the side chain at C7 of the tetrapyrrole, where Chl a has a methyl group and Chl *b* has a formyl group. Chl *a* take part in the reaction of both the photosynthetic reaction centers and the light-harvesting antennae, whereas Chl *b* only occurs in the antenna complexes and can control the photosynthetic antenna size to enable optimal utilization of available light^2–5^. Increasing Chl content is an important approach for improving crop yield by increasing the photosynthetic rate and promoting photoassimilate accumulation, which is also a focus of breeders^6–8^.

Chl is synthesized in 16 steps from glutamyl tRNA in higher plants, and Chl *b* is synthesized by chlorophyllide a oxygenase (CAO) from Chl *a*^2,9^. Recombinant CAO can catalyze the reaction from chlorophyllide a to chlorophyllide b by a two-step oxidation reaction^3^. CAO is considered as the only enzyme responsible for Chl b synthesis, and defective CAO results in Chl b content cut down in *Chlamydomonas reinhardtii*^2^ and *Arabidopsis thaliana*^10,11^. The sequence of CAO is highly conserved from cyanobacteria to higher plants^12–15^. CAO consists of three domains: A, B, and C^16^. The A domain, i.e., the N-terminal conserved sequence, is considered to perceive the existence of Chl *b* and participates in regulating CAO protein levels^17,18^. The C domain includes a [2Fe–2S] Rieske center and mononuclear iron motif that exercise its catalytic activity^12^. The B domain, between the A and C domains, is less conserved, which may be related to the protein stability of CAO^19–21^.

In higher plants, the coding region of most genes (80–85%) is composed of exons and introns^22^. An exon is the part of DNA that is retained after pre-RNA is cut or modified. It appears in the gene sequence of mature RNA and can be translated to protein to perform gene functions. Introns in DNA are transcribed into the pre-RNA, but during RNA splicing, the introns in the transcripts of primary genes are removed, and the exons are connected to produce mature mRNA. Although introns cannot be transcribed and translated into proteins, many studies have found that introns play an important role in alternative splicing and regulation of gene expression^23–25^, especially when the intron is placed close to the 5′ end of the gene^26,27^. He et al.^28^ first showed that the length of intron 1 in *BrMYB2* regulates anthocyanin biosynthesis and controls the development of a purple or white head in Chinese cabbage. Lasin et al.^29^ showed that the two introns of *ATSUC1* are required for gene expression in roots.

Although some leaf color-related genes have been cloned in Chinese cabbage, there is still a difference in the mechanism and characteristics; thus, more leaf color mutant genes need to be cloned. This study completed a mapping of the pale green mutant gene (*Brpgm*) in Chinese cabbage and revealed that a single nucleotide polymorphism exists in *BraA10g007770.3C* (*BrCAO*), resulting in premature translational termination. Based on the identification of phenotypic, genetic, and photosynthetic characteristics of the pale green mutant (*pgm*), it is suggested that *BrCAO* plays a critical role in Chl synthesis and yield in Chinese cabbage.

## Results

### Mutant characteristics identification

**Figure 1:**
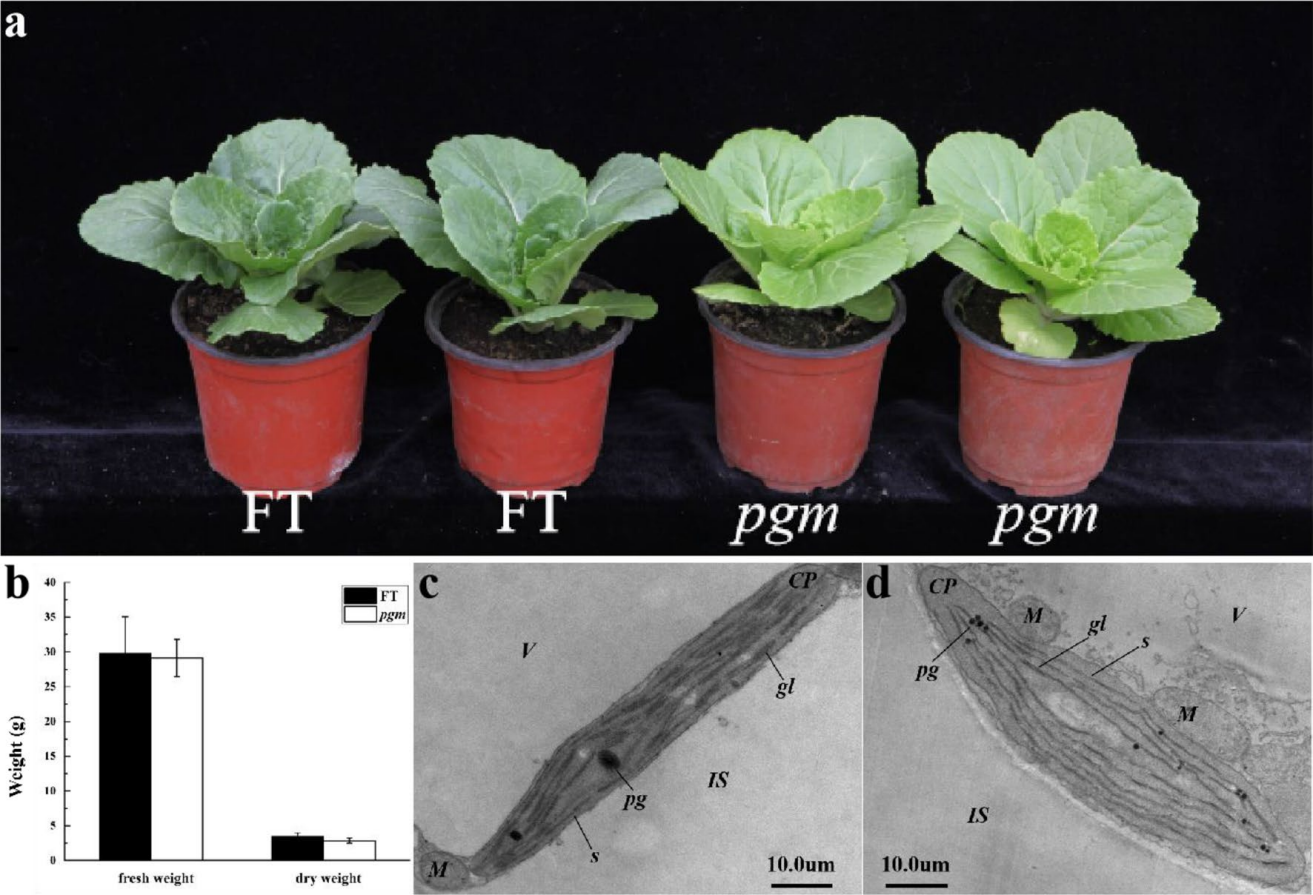
Phenotypic characterization and ultrastructure of chloroplasts of pale green mutant (*pgm*) and wild-type (FT). (**a**) Phenotypic characterization of *pgm* (right) and FT (left) at 6 weeks. (**b**) The fresh weight and dry weight in *pgm* and FT at 6 weeks. (**c**, **d**) Ultrastructure of chloroplasts in FT (**c**) and *pgm* (**d**). Scale is shown at the bottom.

The *pgm* plants appeared evenly pale green and showed normal growth (Fig. 1a). In the vegetative growth and reproductive growth stages, *pgm* and FT were both able to develop normal heading, flowering, and seed setting. Biomass was analyzed by measuring the fresh weight and dry weight of the whole plant at the age of 6 weeks. No significant differences were observed both on fresh weight and dry weight of *pgm* and FT (Fig. 1b).

### Photosynthesis pigment content and chloroplast structure

Compared with FT, *pgm* showed a more significant reduction in both total Chl and Car content (Table 1). In *pgm*, Chl *a*, Chl *b*, and Car, 50.63%, 82.46%, and 59.65% reduction caused a higher level of Chl *a*/*b* and a stable level of Car/Chl, suggesting that the obstruction of the Chl *b* synthesis pathway was greater than that of the Chl *a* synthesis pathway.

**Table 1:** Content of photosynthetic pigments of leaves in pale green mutant (*pgm*) and wild-type (FT).

Materia	Chl *a*	Chl *b*	Chl	Chl *a*/*b*	Car	Car/Chl
FT	13.51 ± 0.40*	4.79 ± 0.29*	18.30 ± 0.69*	2.87 ± 0.07*	4.51 ± 0.29*	0.24 ± 0.01
*pgm*	6.67 ± 0.62	0.84 ± 0.05	7.50 ± 0.66	7.95 ± 0.36	1.82 ± 0.15	0.24 ± 0.01

The t-test with paired comparison was used to test the significant differences between the wild-type FT and pale green mutant (*pgm*). The ‘*’ followed by figures represents significant differences at 5% levels.

To investigate whether the chloroplast development of *pgm* affected, we observed the ultrastructure of chloroplasts in *pgm* and FT using transmission electron microscopy (Fig. 1c,d). The chloroplasts of *pgm* were plump and oval in shape, but the thylakoid lamellae were loose, with more starch grain accumulation. The FT chloroplasts were elongated and elliptic, with well-developed thylakoid membrane systems, large number of grana, tight and orderly arrangements, abundant matrix lamellae, and more stacking.

### Photosynthetic parameters and fluorescence kinetic parameters

The photosynthetic index of FT and *pgm* was measured, and it was found that the *P*_n_ of *pgm* was 6.97% lower than that of FT; however, the decrease was not significant (Table 2). The *G*_s_ and Ts of *pgm* did not change significantly, but the *C*_i_ of *pgm* increased significantly.

**Table 2:** Photosynthetic parameters and fluorescence kinetic parameters of leaves in pale green mutant (*pgm*) and wild-type (FT).

Traits	Characteristics	FT	*pgm*
Photosynthetic parameters	Net photosynthetic rate (Pn, μmol CO_2_ m^−2^ s^−1^)	13.78 ± 0.71	12.82 ± 1.55
	Stomatal conductance (Gs, mol H_2_O m^−2^ s^−1^)	0.4237 ± 0.28	0.2803 ± 0.09
	Intercellular CO_2_ concentration (Ci, μmol CO_2_ mol^−1^)	246.00 ± 28.28	336.00 ± 21.27*
	Transpiration rate (Ts, mmol H_2_O m^−2^ s^−1^)	4.08 ± 0.36	4.87 ± 0.74
Fluorescence kinetic parameters	Minimum fluorescence (F_0_)	0.1067*	0.0550
	Maximum fluorescence (Fm)	0.5783*	0.1697
	Primary photochemical efficiency of PS II (Fv/Fm)	0.8150*	0.6757
	Actual photochemical efficiency of PSII (ΦPSII)	0.4000	0.5350*
	Photochemical quenching (qP)	0.7070	0.9453*
	Non-photochemical quenching (NPQ)	0.6000*	0.1493

The t-test with paired comparison was used to test the significant differences between the wild-type FT and pale green mutant (*pgm*).The ‘*’ followed by figures represents significant differences at 5% levels.

Compared to that of FT, the F_0_, Fm, primary photochemical efficiency of PSII (Fv/Fm), and NPQ of *pgm* were significantly reduced. However, the actual photochemical efficiency of PSII (ΦPSII) and PQ of *pgm* were significantly increased (Table 2); the lack of significant change in the *P*_n_ in *pgm* was possibly attributable to this.

### Inherited characteristic

We constructed genetic populations by FT and *pgm* to figure out the inheritance pattern of *pgm* (Table 3). The inbred offspring of FT (P_1_) and *pgm* (P_2_) showed green and pale green, respectively, proving both mutant and wild-type could be inherited stably. F_1_ (P_1_ × P_2_) and rF_1_ (P_2_ × P_1_) plants showed green color, indicating that the pale green character of *pgm* showed nuclear inheritance and was controlled by recessive nuclear genes. In the F_2_ population, there was character segregation, and the ratio of green plants to pale green plants was 3:1, indicating that the mutation was controlled by one pair of recessive nuclear genes. BC_1_ (F_1_ × P_1_) was green, while in BC_1_ (F_1_ × P_2_), the green plants were separated from the pale green plants by 1:1, which further confirmed the single recessive inheritance of the mutant trait.

**Table 3:** Genetic analysis of *pgm* in Chinese cabbage.

Generation	Green-colored plant	Pale green-colored plant	Total	Segregation ratio	χ^2^ test
P_1_ (FT)	113	0	113		
P_2_ (*pgm*)	0	178	178		
F_1_ (P_1_ × P_2_)	18	0	18		
rF_1_ (P_2_ × P_1_)	25	0	25		
F_2_	63	19	82	3.32:1	0.146 (3:1)
BC_1_ (F_1_ × P_1_)	46	0	46		
BC_1_ (F_1_ × P_2_*)*	20	23	43	0.80:1	0.209 (1:1)

### The *Brpgm* located on chromosome A10 via BSR-seq

**Figure 2:**
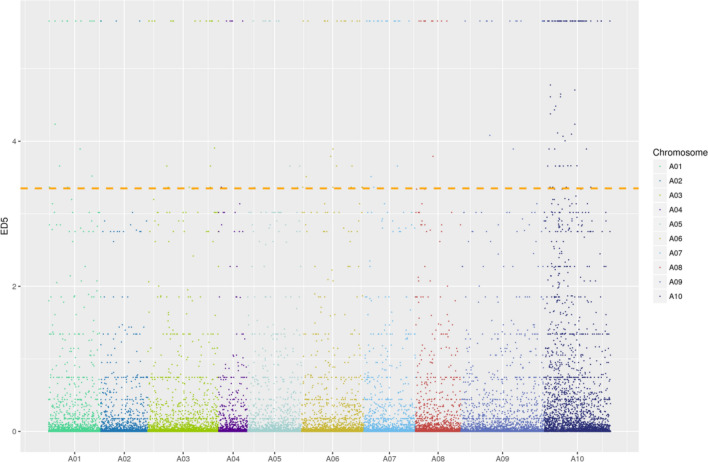
The distribution of ED^5^ values on chromosomes.

Based on the genetic analysis of *pgm*, we constructed a large-scale F_2_ segregated population of *pgm* and K23. The two RNA mixing pools (GP-pool and PGP-pool) were separately constructed by green phenotype and pale green phenotype plants of F_2_ population and sequenced by Illumina for BSR-Seq analysis. Finally, 11,625,396 and 9,526,538 raw reads and 11,558,596 and 9,477,028 clean reads with an average length of 148.51 bp and 148.34 bp, respectively, were obtained (Supplementary Table S1). The clean data were compared with the reference genome, and 80% of the reads of PGP and GP were mapped. Single nucleotide polymorphisms (SNPs) were detected in the GP pool and PGP pool (Supplementary Table S2). SNPs with a coverage depth greater than 3X were screened in the two samples simultaneously, and the Euclidean Distance (ED) of these SNPs was calculated. The ED values of the SNP loci were processed by the fifth power method, ED^5^, to avoid the influence of background noise on the experimental results. A total of 48,827 SNPs were applied to map the ED^5^ values, depending on the distribution of each SNP locus on the chromosome. A statistical peak of ED^5^ was found on chromosome A10, with the top 1% as the threshold, and four correlation intervals were determined (A10: 0.12–2.50 Mb, 3.17–4.49 Mb, 8.56–9.73 Mb, and 10.31–12.51 Mb) (Fig. 2 and Supplementary Table S3).

Based on the above information, SSR primers were designed in four target regions, and the polymorphism of primers was screened by mutant *pgm* and K23 for further experiments (Supplementary Fig. S1a). Primer sequence information is detailed in Supplementary Table S4. We identified 30 individuals with a pale green phenotype in the F_2_ population as a small group to validate BSR-Seq results. The results showed that SSR11-45 and SSR1-10 on chromosome A10 were linked to *Brpgm* and located on both sides of *Brpgm*, according to linkage analysis (Supplementary Fig. S1b,c). These results illustrated that the *Brpgm* gene was mapped on chromosome A10 between SSR11-45 and SSR1-10.

### Mapping of the *Brpgm* to a 4499.6 Kb interval based on linkage analysis

**Figure 3:**
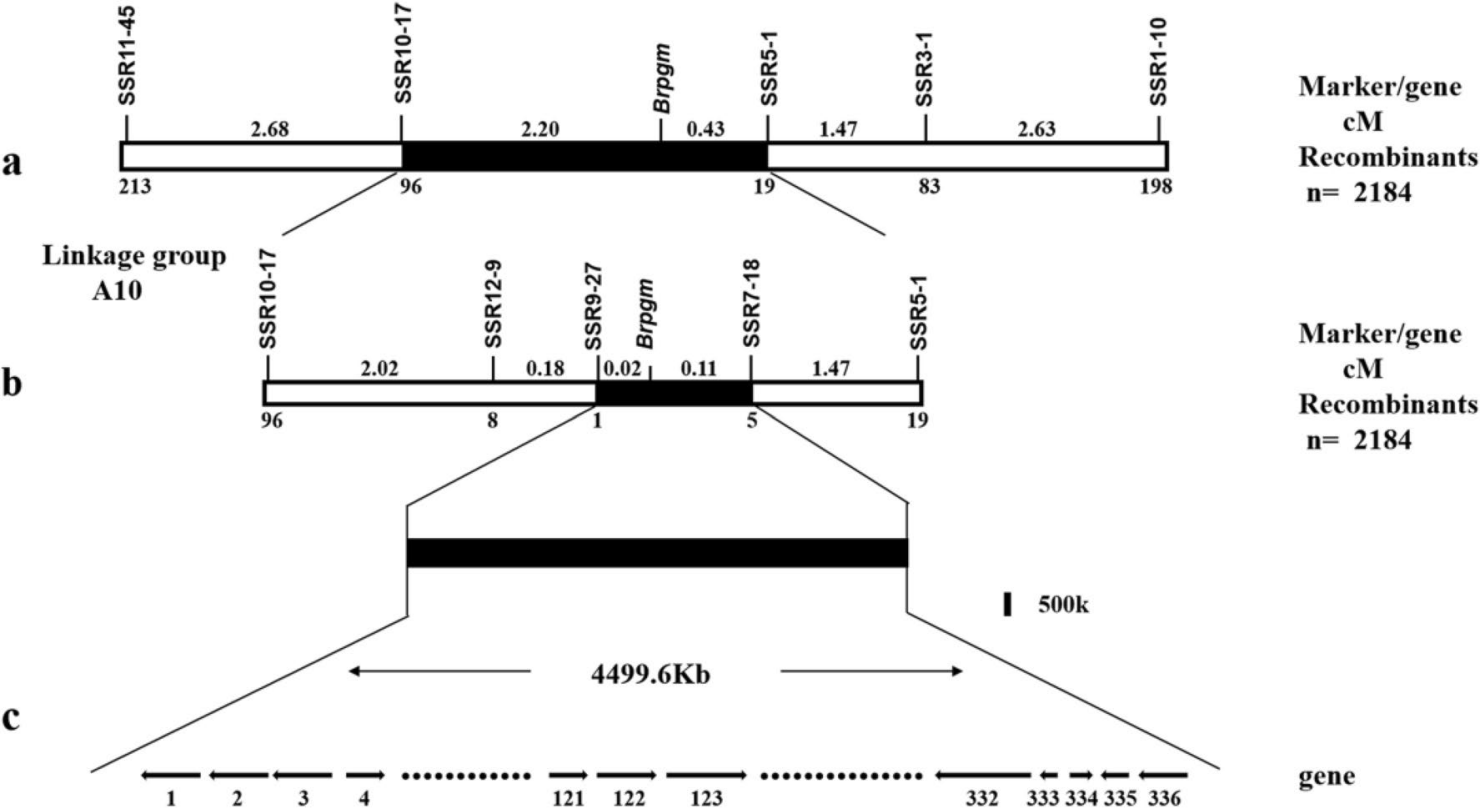
Genetic and physical maps of the *Brpgm* gene locus in Brassica rapa. (**a**) The mutant gene *Brpgm* was initially located between the molecular markers SSR10-17 and SSR5-1 in the A10 linkage group, with genetic distances of 2.20 and 0.43 cM, respectively. (**b**) The mutation gene *Brpgm* was located between SS9-27 and SSR 7–18 with genetic distances of 0.02 and 0.11 cM, respectively. (**c**) The physical size of the candidate region was 4499.6 Kb, containing 336 candidate genes.

Using 2184 F_2_ individuals with a pale green phenotype, we developed new primers and polymorphic markers that were screened and amplified, and calculated the recombination rate. Finally, the markers SSR10-17, SSR12-9, SSR9-27, SSR7-18, SSR5-1, and SSR3-1 were closely linked to the target gene. SSR3-1, SSR5-1, SSR7-18, and SSR1-10 were on the same side, while SSR10-17, SSR12-9, SSR9-27, and SSR11-45 were on the other side of the target gene. The genetic distances between the mutant gene and SSR 7–18 and SSR 9–27 were 0.11 and 0.02 cM (Fig. 3b), respectively. The mutation gene *Brpgm* is located in a physical range of 4499.6 Kb (Fig. 3c), in which there are 336 genes (Supplementary Table S5).

### Candidate gene prediction by whole-genome resequencing

**Figure 4:**
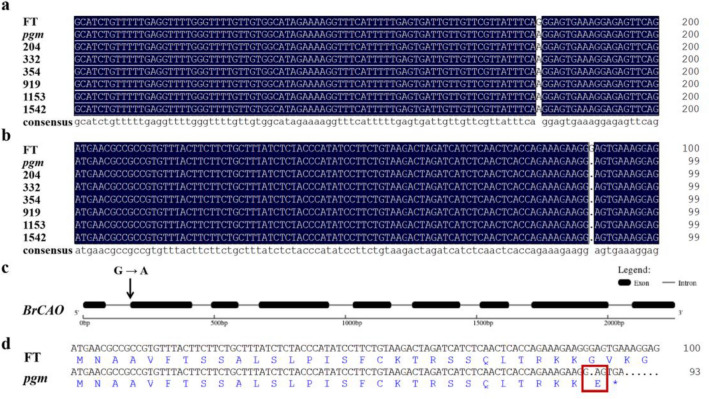
Sequence alignment and gene structure. (**a**) DNA sequence alignment of wild-type (FT), pale green mutant (*pgm*), and F_2_ recombinant individuals: 204, 332, 354, 919, 1153, and 1542. (**b**) cDNA sequence alignment of FT, *pgm*, and F_2_ recombinant individuals. (**c**) Gene structure of *BrCAO*. (**d**) BrCAO protein sequence of FT and *pgm*. The red box indicates the mutation position.

Limited by the size of the mapping population and the number of recombined individuals, the candidate region could not be further narrowed by linkage analysis. Therefore, whole-genome resequencing was carried out in FT and *pgm* lines to detect the mutation sites. Sequencing results showed that just one homozygous non-synonymous SNP was located in the candidate region (Table 4), which consistent with the EMS mutagenesis principle (variations in G-to-A and C-to-T)^30^ and located on *BraA10g007770.3C*. The gene annotation indicated that *BraA10g007770.3C* (*BrCAO*) encodes chlorophyllide a oxygenase (CAO), which converts chlorophyllide a to chlorophyllide b by catalyzing two successive hydroxylations at the 7-methyl group of chlorophyllide a. Clone sequencing revealed that the 180th nucleotide of full length was changed from G to A, and the 88th nucleotide of CDS was deleted (Fig. 4a,b). Based on the gene information (Fig. 4c and Supplementary Table S6), we found that the SNP was located in the last position of the first intron, which changed the splicing mode and resulted in the deletion of the first nucleotide in the second exon. Sequencing of six recombinant individuals of the two most recent markers verified that the SNP was co-isolated with the mutant phenotype (Fig. 4a,b). The deletion of G in CDS caused a frameshift mutation, leading to the early termination of protein translation (Fig. 4d).

**Table 4:** Single nucleotide polymorphism (SNP) in the candidate region between wild-type (FT) and pale green mutant (*pgm*).

Gene ID	Mutation	Chromosome: position	FT-genotype	*pgm*-genotype	Annotation
*BraA10g007770.3C*	Splicing	A10: 5,140,932	G	A	Encodes chlorophyllide a oxygenase (CAO)

### Expression analysis and enzyme assay

**Figure 5:**
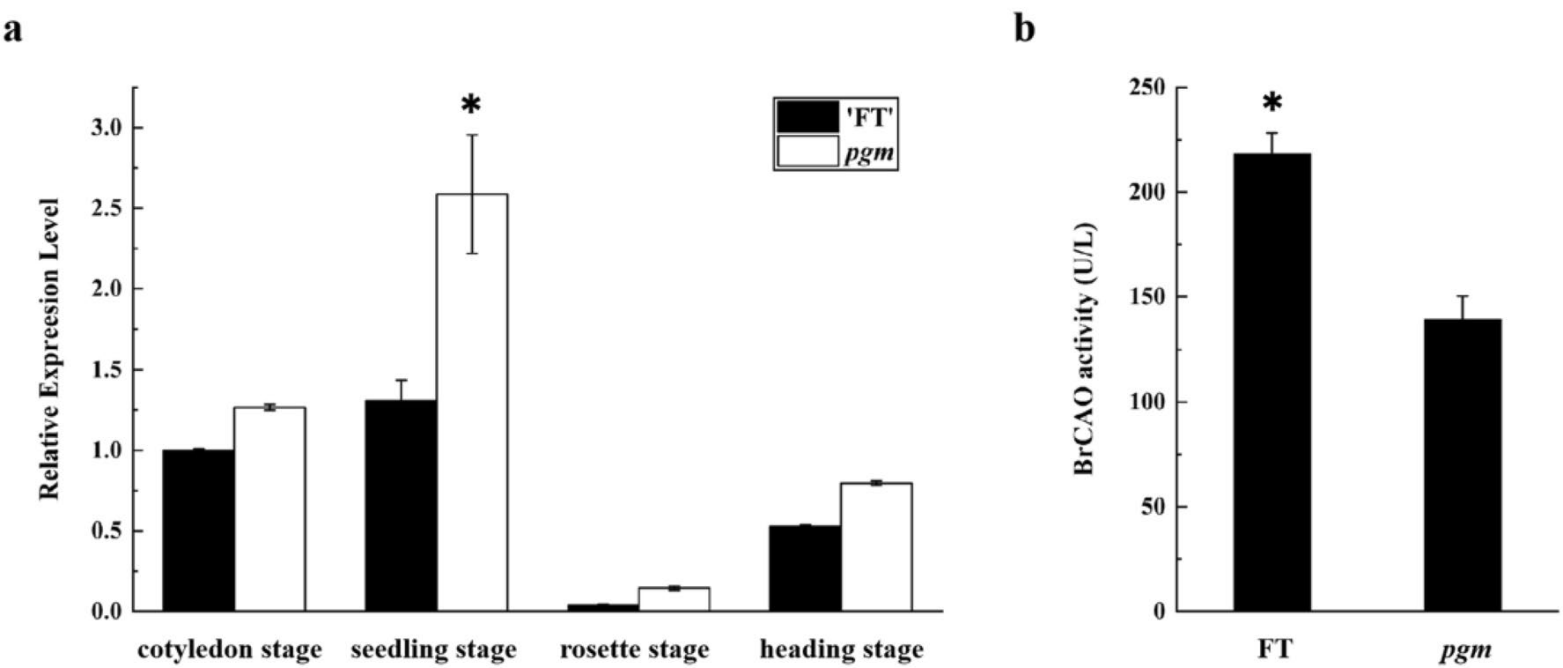
Expression analysis of *BrCAO* and enzyme assay of BrCAO. (**a**) The expression pattern of *BrCAO* in leaves of four stages (cotyledon, seedling, rosette, and heading stage) in pale green mutant (*pgm*) and wild-type (FT). (**b**) The CAO activity in *pgm* and FT. U/L = activity unit. The ‘*’ represents significant differences at 5% levels.

The expression pattern of *BrCAO* in leaves was determined using qRT-PCR. The results showed that the expression of *BrCAO* in *pgm* was upregulated in four stages (cotyledon, seedling, rosette, and heading stages), notably in the seedling stage (Fig. 5a). In the seedling stage, the activity of CAO was determined by ELISA and the result showed that the BrCAO activity was significantly lower in *pgm* than in FT (Fig. 5b).

## Discussion

Leaf color mutants are usually related to Chl content, including the regulatory network of genes involved in Chl biosynthesis, degradation, and chloroplast development. In this study, we isolated *pgm*, which showed a pale green phenotype and normal growth (Fig. 1). Based on BSR-Seq, we developed two SSR markers, SSR9-27 and SSR7-18, which are closely linked to *Brpgm* on chromosome A10 (Figs. 2 and 3). Although the genetic distance between SSR9-27 and SSR7-18 was minimal, there was a large physical distance between the two markers, containing 336 candidate genes. After many rounds of expanding this group, we used 2184 recessive individuals to map the cause gene, but a low recombination rate appeared in the localization interval. The location interval (A10: 3,654,958–8,154,544) was located in the centromere interval (A10: 5,449,476–8,087,520) of the A10 chromosome^31^. Previous studies have found that it is difficult to map by centromere intervals because of the high content of repetitive sequences and low recombination frequency^32–36^. Therefore, we think that the larger and more difficult-to-narrow physical distance of the *Brpgm* candidate region is related to its location near the centromere.

Whole-genome resequencing further detected the SNP in the candidate region between FT and *pgm* lines and screened only one homozygous non-synonymous SNP located on *BraA10G007770.3C* (*BrCAO*). Parents and F_2_ recombinant individual sequencing verified that this SNP co-separated with the pale green phenotype. *BrCAO* encodes chlorophyllide a oxygenase (CAO), which is the only essential enzymatic step for Chl b formation^3^. Tanaka et al.^2^ and Espineda et al.^10^ characterized *ATCAO* mutants in *Arabidopsis thaliana*, which have reduced levels of Chl *b* and higher *AtCAO* mRNA levels. Lee et al.^4^ identified Line 1C-039-43, whose the first intron of *OsCAO1* existed a insertion, which was deficient in Chl *b*, producing pale green leaves. In our study, *pgm* had lower chlorophyll content with a higher Cha/b value and lower BrCAO activity with higher *BrCAO* mRNA levels. Mueller et al.^15^ by multiple independent alleles validated the candidate gene of a light green chlorina phenotype (due to the reduction in Chl *b*) as *HvCAO*. Pattanayak et al.^37^ revealed that overexpression of *CAO* in tobacco could increase chlorophyll (Chl) b biosynthesis and decrease the Chl *a*/*b* ratio.

RNA splicing is essential for the development and evolution of organisms, and the key to splicing recognition depends on the determination of the splicing sites. The splicing sites of pre-mRNA in eukaryotic cells mostly follow the GU-AG rule; that is, the base groups of intron 5′ end (donor site) and 3′ terminal (acceptor site) are almost GU and AG^22,38^. In this study, the 180th nucleotide of *BrCAO* full length varied from G to A in *pgm*, which is located in the last position of the first intron. Although this SNP is located in the intron, it leads to the deletion of the 88th nucleotide in CDS, located in the first nucleotide of the second exon. This result may be associated with RNA splicing. The 180th nucleotide of full length changing from G to A, resulting in the last two positions of the first intron (RNA splice sites) changed from AG to AA. The first nucleotide (G) of the second exon and the last nucleotide (A) of the first intron forms a new RNA splice site AG, which results in the deletion of the first nucleotide (G) of the second exon in CDS. The deletion of nucleotide G in CDS resulted in a frameshift mutation, leading to the early termination of protein translation.

As the main product of Chinese cabbage plants, the leaves have always been of concern. In this study, *pgm* appeared to be stably pale green and possessed a lower Chl content. Unlike most yellow leaf mutants, the deficiency of Chl content in *pgm* did not lead to weak growth and weight reduction but regulated the color of leaves. This result may be related to the fact that P_n_ was not significantly different and due to higher PQ values with lower NPQ values. In *pgm*, most of the light energy absorbed by the PSII antenna pigment is used for photochemical reaction electron transfer. Only a small part cannot be used for photosynthetic electron transfer but is dissipated in the form of heat. Despite the decrease in chlorophyll content, the net photosynthetic efficiency was stable and showed a younger color in *pgm*. Therefore, *pgm* could be considered a new germplasm for leaf color improvement without affecting yield.

In conclusion, we identified *pgm* created by EMS mutagenesis in Chinese cabbage. BSR-Seq and linkage analysis mapped *Brpgm* in the centromere interval of chromosome A10. Whole-genome resequencing analysis predicted that the target gene was *BrCAO*. The mutation of *BrCAO* in *pgm* occurred at the splice site of the first intron, which resulted in the early termination of the protein translated by *BrCAO*. *pgm* showed a pale green phenotype and possessed lower Chl content with a higher Chl a/b ratio, imperfect chloroplast structure, and lower NPQ. *P*_*n*_ and biomass of *pgm* was not significantly altered, which suggested that *pgm* may be used for color improvement in Chinese cabbage breeding.

## Materials and methods

### Plant materials

The wild-type (FT), doubled haploid (DH) line of Chinese cabbage, was used as the wild type and test materials in ethyl methane sulfonate (EMS) mutagenesis. The *pgm* was identified in the mutant populations. The Pak choi inbred line, “K23” with deep green leaves was used to construct the segregating population with *pgm*. All materials were grown and provided by Shenyang Agricultural University, Shenyang, China.

### Pigment content measurement

Chl and carotenoid (Car) content were determined using a DU 800 UV/Vis Spectrophotometer (Beckman Coulter, La Brea, CA, USA) according to the method outlined by Arnon^39^, with some modifications. The fifth leaves of 6-week-old plant were harvested and submerged in 80% acetone under dark conditions for 24 h. The extracts were measured at 663, 645, and 470 nm. Pigment concentrations were calculated as described by Holm^40^. Three plants were measured per treatment, and each sample carried out three times repeats.

### Determination of photosynthetic parameters

At the age of 6 weeks, the fifth leaves were selected to determine the photosynthetic parameters using a portable photosynthetic system (CIRAS-2, PP Systems, USA). Measurements were recorded for three individual plants per treatment at a sunny day. Data was automatically recorded until a steady net photosynthetic rate (*P*_*n*_) was attained. The photosynthetic parameters consisted of *P*_*n*_, stomatal conductance (*G*_*s*_), intercellular CO_2_ concentration (*C*_*i*_), and transpiration rate (*E*).

### Analysis of Chl fluorescence kinetics

At approximately six-weeks-old, FT and *pgm* plants with the same growth were selected to measure fluorescence parameters using a Chl fluorescence imaging system (IMAGING-PAM, Walz, Germany), which is a platform instrument of the facility of Horticulture at the College of Horticulture. On a sunny morning, the plants were kept away from light for 20 min. Then, the fifth true leaf of the plants were removed and placed in the instrument. The pulse intensity was set to 4500 μmol m^−2^ s^−1^, the pulse time was set to 0.8 s. Then the related Chl fluorescence kinetics parameters were determined and recorded. Three biological repeats were identified in each material.

### Transmission electron microscopy analysis

The same leaf parts of the 6-week-old plants were cut into 2 × 6 mm pieces, pre-fixed in 3% (w/v) glutaraldehyde and stored overnight at 4 °C. After rinse with 1% phosphoric acid buffer, the samples were fixed with 1% osmium acid for 2 h. The following procedures were carried out as described by Zhao et al.^41^.

### Genetic analysis

FT (P_1_) and *pgm* (P_2_) lines were crossed and produced F_1_ (P_1_ × P_2_) and rF_1_ (P_2_ × P_1_). F_1_ plants were self-pollinated to produce an F_2_ population. The BC_1_ populations were derived by the backcrosses of FT and *pgm* lines, respectively, with F_1_. The phenotype characterization and segregation ratio of each generation (P_1_, P_2_, F_1_, rF_1_, F_2_, and BC_1_) were recorded and analyzed using the χ^2^ test. All measurements in the experiments were analyzed using a random design.

### Bulked segregant RNA-sequencing (BSR-seq)

To map the mutant gene *Brpgm* of the *pgm* line, we used K23 and the *pgm* line to construct the F_2_ population. In the F_2_ mapping population, 50 green phenotype plants and 50 pale green phenotype plants with the same growth were selected to extract total RNA. The green phenotype RNA mixing pool (GP-Pool) and pale green phenotype RNA mixing pool (PGP-Pool) were constructed by mixing green phenotype and pale green phenotype plant samples, respectively. RNA was extracted using a plant total RNA extraction kit (Tiangen, Beijing, China), following the manufacturer’s procedure. BSR-Seq was performed and analyzed as described by Zhao et al.^42^.

### DNA extraction and PCR amplification

Total genomic DNA was extracted from fresh leaves using the CTAB method according Murray and Thompson^43^, with minor modifications. A 10 μl system was used as the PCR amplification reaction system, and the PCR procedure was as follows: pre-denaturation at 95 °C for 5 min, one cycle; denaturation at 95 °C for 30 s; renaturation at 56 °C for 30 s; extension at 72 °C for 1 min, 35 cycles; final extension at 72 °C for 5 min.

### SSR marker analysis, linkage analysis, and genetic map construction

Based on the location interval of BSR-Seq, the genome sequence information was downloaded from the Brassica database (http://brassicadb.org/brad/datasets/pub/Genomes/Brassica_rapa/V3.0/). Primer Premier 5.0 software (Premier Inc., Charlotte, USA) was used to design primers. Polyacrylamide gel electrophoresis was used to screen the primers with polymorphisms between the parents. Then, linkage analysis was performed using the polymorphic markers in F_2_ individuals with a pale green phenotype. Segregation data were used to construct a linkage map of the F_2_ population using Join Map 4.0^44^. The genetic map distances (cM) were calculated according to the method of Kosambi^45^.

### Whole-genome resequencing

The genomic DNA of FT and *pgm* were extracted using DNA Secure Plant Kit (Tiangen, Beijing, China) for whole-genome resequencing. A DNA library with 400 bp of insert size was constructed and sequenced using next-generation sequencing and Illumina HiSeq paired-end sequencing (Illumina, San Diego, USA). The raw data were analyzed after removal of joint contamination, quality filtering, and length filtering to generate clean data. The BWAMEM program was used to map the filtered clean data to the reference genome (http://brassicadb.org/brad/datasets/pub/Genomes/Brassica_rapa/V3.0/). GATK software^46^ and ANNOVAR software^47^ were used to extract SNPs (single nucleotide polymorphism) and annotate SNPs, respectively.

### Clone sequencing

The full-length and CDS of *BrCAO* were amplified using FL-BrCAO primers and CDS-BrCAO primers, respectively (Supplementary Table S7). PCR products were purified and ligated to the pGEM-T Easy Vector (Promega, USA). The vectors were transformed into competent *E. coli* cells. After culturing, plasmids were extracted and sequenced using GENEWIZ (Suzhou, China). Sequencing data were analyzed using DNAMAN V6 software (Lynnon BioSoft, Canada).

### Total RNA extraction and gene expressive analysis

Total RNA samples were extracted from fresh leaves of different stages (cotyledon, seedling, rosette, and heading stages) using a plant total RNA extraction kit (Tiangen, Beijing, China). cDNA was synthesized using FastQuant RT Super Mix 13 (Tiangen, Beijing, China) Quantitative real-time PCR (qRT-PCR) amplification was carried out in QuantStudio 6 (Life Technologies, California, USA) using SYBR Green PCR Master Mix (Takara Bio Inc., Kusatsu, Japan) in a 20 μl reaction mixture. Gene-specific primers were designed using Primer Premier 5.0, and the *ACTIN* gene was used as the internal control (Supplementary Table S8). The qRT-PCR amplification reaction system and procedure was described as Huang et al.^48^.

### Enzyme activity assay

An enzyme-linked immunosorbent assay (ELISA) kit (Meimian Industrial Co., Ltd., Jiangsu, China) was used in accordance with the manufacturer’s instructions to determine the activity of CAO. Leaves of six-week-old plants (0.2 g FW) were homogenized in phosphate buffered saline (PH7.4). The supernatant was obtained by centrifugation at 12,000×*g* and used for the analysis. The experimental process was conducted according to the manufacturer’s instructions for ELISA kits.

### Statistical analysis

The t-test was used to analyze the significant differences at a significance level of 0.05.

### Ethical approval

The study was performed in accordance with relevant guidelines and regulations.

## Supplementary Information

Supplementary Information.

## Data Availability

The research data underpinning this publication can be accessed at https://dataview.ncbi.nlm.nih.gov/object/34036491 and https://dataview.ncbi.nlm.nih.gov/object/34036030.
