# Supplementary material for: Identification of a biomass unaffected pale green mutant gene in Chinese cabbage (Brassica rapa L. ssp. pekinensis)
Source: Sci Rep. 2022 May 11;12:7731. doi: 10.1038/s41598-022-11825-1 (PMC9095832; doi:10.1038/s41598-022-11825-1)
Supplement: Supplementary file 1 — Supplementary Information. [file 41598_2022_11825_MOESM1_ESM.docx]

Figure S1. Polymorphism screened by the SSR primers between the two parents (a) and amplification of SSR11-45 (b) and SSR1-10 (c) in F2 plants.


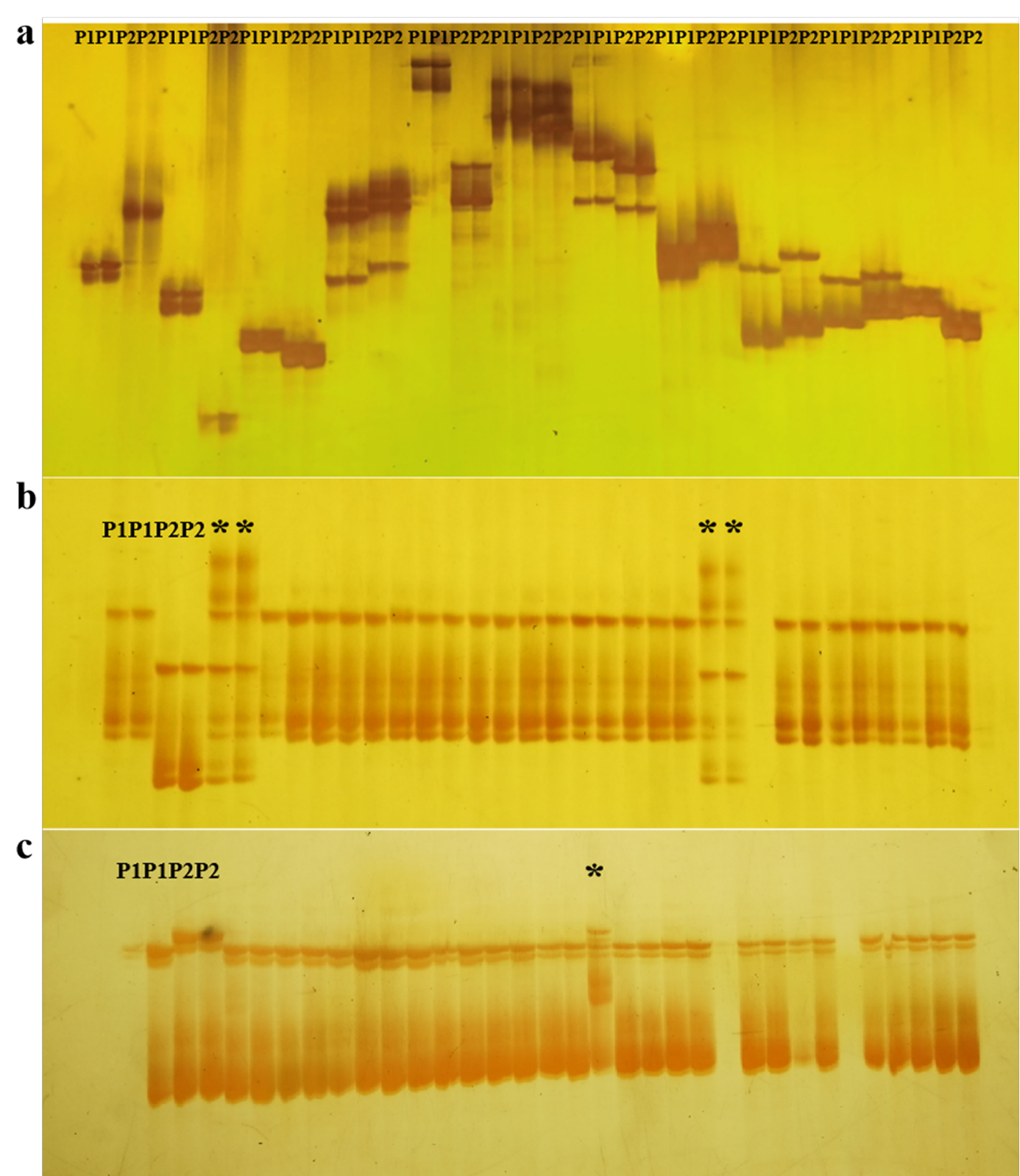


P1: *pgm*；P2: K23; *: recombinant individuals

Table S1 Quality statistics of raw data and clean data

| Data | Sample | length(bp) | Raw reads | Q20(%) | Q30(%) | GC(%) | N(ppm) |
| --- | --- | --- | --- | --- | --- | --- | --- |
| Raw data | GP-pool | 150.00 | 11,625,396 | 96.84 | 92.39 | 48.50 | 10.07 |
|  | PGP-pool | 150.00 | 9,526,538 | 96.94 | 92.60 | 48.13 | 10.08 |
| Clean data | GP-pool | 148.51 | 11,558,596 | 97.21 | 92.91 | 48.54 | 0.84 |
|  | PGP-pool | 148.34 | 9,477,028 | 97.29 | 97.29 | 48.17 | 0.81 |

Table S2 Reads mapped to the reference genome

| Sample | Total reads | Total mapped | Multiple mapped | Unique match | Non-splice reads | Splice reads |
| --- | --- | --- | --- | --- | --- | --- |
| GP-pool | 11,558,596 | 9,287,977 (80%) | 1,215,093 | 8,072,884 | 4,706,753 | 3,366,131 |
| PGP-pool | 9,477,028 | 7,590,775 (80%) | 947,734 | 6,643,041 | 3,853,521 | 2,789,520 |

Table S3 The localization of chromosomal loci related to mutant trait

| chromosome | start position | end position | interval length(bp) | Total_Gene |
| --- | --- | --- | --- | --- |
| A10 | 11,726 | 2,497,916 | 2,486,190 | 382 |
| A10 | 3,172,536 | 4,490,115 | 1,317,579 | 180 |
| A10 | 8,560,112 | 9,730,110 | 1,169,998 | 201 |
| A10 | 10,307,158 | 12,513,192 | 2,206,034 | 433 |

Table S4 Primer sequences of SSR markers

| Marker | Forward sequence (5’-3’) | Reverse sequence (5’-3’) |
| --- | --- | --- |
| SSR11-45 | GGAAAAGACGAAAATACCCTCA | AAAAGCCAAATGAACAATCCC |
| SSR10-17 | TTTTAACTAGGGCTGGATGAGA | AAACCAAGAGATTCGGACAAG |
| SSR12-9 | CGGGAGAAGGAGAGGTGATT | ATTGCTGGTGGGGCCA |
| SSR9-27 | AATTGAAATGTGTCTTAGTCGCA | ATTAGAAAAGTGAATAGCATTTTAGTAAA |
| SSR7-18 | TGAACTTGTGGGGTGAATATG | AGGCAATCCCGTTTCTTAAT |
| SSR5-1 | TCTTCCTCGTACTCATTGTTTGT | TCAAGGTAAAATTTGATTAGCGT |
| SSR3-1 | AATTCTTTGACGAAAAACACAAG | ACCTTTCATGCAATATTATTACTCTT |
| SSR1-10 | CATCATCAGTCTCCATACCATCAT | CTCGTCTCTTCAACTTCTGTGTTT |

Table S5 Prediction of candidate genes within the gene-mapped region on chromosome A10

| Gene | Start | End | Gene Annotations (BLASTX to Arabidopsis thaliana) | E value |
| --- | --- | --- | --- | --- |
| BraA10g006560.3C | 3,652,945 | 3,655,339 | RNA binding protein | 3.00E-51 |
| BraA10g006570.3C | 3,662,001 | 3,664,391 | RNA binding protein | 3.00E-39 |
| BraA10g006580.3C | 3,670,997 | 3,673,392 | RNA binding protein | 5.00E-50 |
| BraA10g006590.3C | 3,677,437 | 3,678,664 | Succinate dehydrogenase subunit 5, mitochondrial | 1.00E-140 |
| BraA10g006600.3C | 3,678,900 | 3,679,572 | Uncharacterized protein | 1.00E-64 |
| BraA10g006610.3C | 3,687,848 | 3,688,141 | Calmodulin binding | 0.002 |
| BraA10g006620.3C | 3,710,033 | 3,712,053 | Probable protein phosphatase 2C 12 | 0.0 |
| BraA10g006630.3C | 3,717,276 | 3,719,855 | 3beta-hydroxysteroid-dehydrogenase/decarboxylase | 0.0 |
| BraA10g006640.3C | 3,731,339 | 3,737,046 | Uncharacterized protein LOC103834526 | 0.023 |
| BraA10g006650.3C | 3,737,618 | 3,739,499 | Cystathionine beta-synthase (CBS) family protein | 1.00E-150 |
| BraA10g006660.3C | 3,739,766 | 3,741,232 | Tubby-like F-box protein 6 | 1.00E-176 |
| BraA10g006670.3C | 3,805,674 | 3,806,081 | Plant self-incompatibility protein S1 family | 2.00E-28 |
| BraA10g006680.3C | 3,811,832 | 3,812,302 | Aminopeptidase | 0.24 |
| BraA10g006690.3C | 3,822,081 | 3,826,606 | Formin-like protein 7 | 0.019 |
| BraA10g006700.3C | 3,836,613 | 3,837,086 | Proteolysis involved in cellular protein catabolic process | 3.00E-46 |
| BraA10g006710.3C | 3,845,339 | 3,846,429 | Calcium-dependent protein kinase 6 | 0.1 |
| BraA10g006720.3C | 3,846,476 | 3,846,700 | Zinc finger CCCH domain-containing protein 58 | 4.00E-04 |
| BraA10g006730.3C | 3,848,431 | 3,850,403 | Metal ion transmembrane transporter | 0.0 |
| BraA10g006740.3C | 3,850,757 | 3,852,385 | Regulation of cell cycle | 0.0 |
| BraA10g006750.3C | 3,853,170 | 3,857,113 | Hypothetical protein | 0.3 |
| BraA10g006760.3C | 3,857,611 | 3,858,222 | Protein GAMETE expresse 2 | 0.25 |
| BraA10g006770.3C | 3,859,242 | 3,859,613 | Phospho acetyl glucosamine mutase | 0.6 |
| BraA10g006780.3C | 3,859,967 | 3,860,644 | Hypothetical protein | 4.4 |
| BraA10g006790.3C | 3,874,537 | 3,876,232 | Cyclin-A3-2 | 0.0 |
| BraA10g006800.3C | 3,877,017 | 3,877,517 | WPP domain-containing protein 2 | 1.00E-30 |
| BraA10g006810.3C | 3,881,110 | 3,881,845 | Hypothetical protein | 0.014 |
| BraA10g006820.3C | 3,883,335 | 3,887,951 | Hypothetical protein | 0.003 |
| BraA10g006830.3C | 3,896,002 | 3,898,247 | Cysteine-type peptidase activity | 0.0 |
| BraA10g006840.3C | 3,914,408 | 3,915,985 | Intrinsic thylakoid membrane protein | 1.00E-43 |
| BraA10g006850.3C | 3,927,841 | 3,928,819 | Late embryogenesis abundant (LEA) protein | 0.004 |
| BraA10g006860.3C | 3,934,946 | 3,936,523 | Intrinsic thylakoid membrane protein | 1.00E-43 |
| BraA10g006870.3C | 3,950,140 | 3,950,349 | Alcohol dehydrogenase | 0.021 |
| BraA10g006880.3C | 3,950,501 | 3,950,896 | Serine/threonine-protein kinase KIPK1 | 0.064 |
| BraA10g006890.3C | 3,953,364 | 3,954,431 | Heat stress transcription factor B-4 | 1.00E-100 |
| BraA10g006900.3C | 3,985,479 | 3,989,958 | Hypothetical protein | 0.022 |
| BraA10g006910.3C | 3,991,892 | 3,993,081 | Alanine--tRNA ligase, chloroplastic/mitochondrial | 0.46 |
| BraA10g006920.3C | 3,994,397 | 3,995,764 | Transcription initiation factor TFIID subunit | 2.9 |
| BraA10g006930.3C | 4,009,145 | 4,014,762 | S-ribonuclease binding protein | 1.00E-124 |
| BraA10g006940.3C | 4,016,894 | 4,018,073 | Transcription factor | 0.029 |
| BraA10g006950.3C | 4,020,865 | 4,022,533 | Transmembrane protein | 0.0 |
| BraA10g006960.3C | 4,024,978 | 4,029,232 | Hypothetical protein | 0.3 |
| BraA10g006970.3C | 4,037,249 | 4,038,244 | Hypothetical protein | 0.1 |
| BraA10g006980.3C | 4,051,611 | 4,053,961 | Weak chloroplast movement under blue light-like protein (DUF827) | 1.00E-128 |
| BraA10g006990.3C | 4,087,885 | 4,089,755 | ABF2 abscisic acid responsive elements-binding factor 2 | 2.00E-71 |
| BraA10g007000.3C | 4,090,871 | 4,092,459 | Hypothetical protein | 2.00E-90 |
| BraA10g007010.3C | 4,092,867 | 4,093,400 | Hypothetical protein | 0.22 |
| BraA10g007020.3C | 4,093,931 | 4,094,788 | Hypothetical protein | 0.23 |
| BraA10g007030.3C | 4,096,845 | 4,099,468 | methylguanosine RNA capping | 0.0 |
| BraA10g007040.3C | 4,108,538 | 4,108,867 | Hypothetical protein | 0.034 |
| BraA10g007050.3C | 4,110,570 | 4,112,291 | Hypothetical protein | 0.026 |
| BraA10g007060.3C | 4,115,415 | 4,118,608 | Calcium-dependent lipid-binding (CaLB domain) family protein | 0.077 |
| BraA10g007070.3C | 4,137,039 | 4,140,630 | PRLI-interacting factor K | 3.00E-22 |
| BraA10g007080.3C | 4,143,827 | 4,144,585 | Hypothetical protein | 4.00E-60 |
| BraA10g007090.3C | 4,165,689 | 4,169,031 | Zinc ion binding | 0.022 |
| BraA10g007100.3C | 4,171,578 | 4,173,783 | MATE efflux protein-related | 0.012 |
| BraA10g007110.3C | 4,176,568 | 4,177,239 | Ubiquitin-protein ligase | 0.40 |
| BraA10g007120.3C | 4,190,474 | 4,190,701 | Transferring phosphorus-containing groups | 0.35 |
| BraA10g007130.3C | 4,192,673 | 4,193,086 | Hypothetical protein | 0.043 |
| BraA10g007140.3C | 4,210,408 | 4,215,119 | Lipid metabolic process | 0.0 |
| BraA10g007150.3C | 4246751 | 4266289 | Triacylglycerol lipase-like | 0 |
| BraA10g007160.3C | 4269820 | 4271213 | ARABIDOPSIS ISOCHORISMATE SYNTHASE 1, ATICS1, | 3.00E-40 |
| BraA10g007170.3C | 4274091 | 4275250 | Nucleotidylyl transferase superfamily protein | 2.00E-21 |
| BraA10g007180.3C | 4285610 | 4286698 | Encodes one of four UDP-glucose dehydrogenase UGD) genes | 0.074 |
| BraA10g007190.3C | 4295681 | 4299096 | RING/U-box superfamily protein; | 8.00E-144 |
| BraA10g007200.3C | 4302677 | 4303737 | Nucleic acid-binding proteins superfamily | 0.002 |
| BraA10g007210.3C | 4326621 | 4327062 | Encodes a cytosolic thioredoxin | 1.00E-101 |
| BraA10g007220.3C | 4327224 | 4331035 | Beta-galactosidase 5 | 0 |
| BraA10g007230.3C | 4347251 | 4348986 | Tetrapyrrole (Corrin/Porphyrin) Methylase | 0 |
| BraA10g007240.3C | 4359690 | 4361252 | Member of ubiquitin-conjugating E2-proteins | 8.00E-97 |
| BraA10g007250.3C | 4365211 | 4365804 | Auxin response factor 18 | 0 |
| BraA10g007260.3C | 4372585 | 4374124 | Encodes a class III peroxidase that is genetically redundant with PRX40 | 0 |
| BraA10g007270.3C | 4374893 | 4376044 | SNARE associated Golgi protein family | 0 |
| BraA10g007280.3C | 4390056 | 4391478 | Transmembrane protein | 0 |
| BraA10g007290.3C | 4396589 | 4403798 | Binds the carboxyl-terminal domain (CTD) of the largest subunit of RNA polymerase II and functions as a scaffold for RNA processing machineries. | 0 |
| BraA10g007300.3C | 4404473 | 4404739 | CORD2 is a member of a novel and plant specific family of microtubule associated proteins | 2.00E-21 |
| BraA10g007310.3C | 4460121 | 4460833 | Replication factor-A protein 1-like protein | 0.11 |
| BraA10g007320.3C | 4471654 | 4471938 | Encodes Tunicamycin Induced 1(TIN1), a plant-speci‑c ER stress-inducible protein | 0.77 |
| BraA10g007330.3C | 4475705 | 4475890 | Encodes a member of heat shock protein 70 family | 1.7 |
| BraA10g007340.3C | 4477836 | 4480379 | Encodes Siliques Are Red 1 (SIAR1) | 0 |
| BraA10g007350.3C | 4503254 | 4503588 | Encodes a vacuolar glucose exporter | 5.00E-32 |
| BraA10g007360.3C | 4506045 | 4506308 | A member of the cytochrome P450 family | 0.2 |
| BraA10g007370.3C | 4507431 | 4508065 | Encodes a K homology (KH) domain-containing | 0.037 |
| BraA10g007380.3C | 4592053 | 4594095 | Peptidase M20/M25/M40 family protein | 0 |
| BraA10g007390.3C | 4608435 | 4608878 | Encodes a receptor-like protein kinase that is expressed in roots. | 5.28 |
| BraA10g007400.3C | 4625058 | 4626024 | Ribonuclease II family protein | 2.00E-21 |
| BraA10g007410.3C | 4246751 | 4266289 | Encodes a nuclear-localized member of the DREB subfamily A-5 of ERF/AP2 transcription factor family | 4.00E-67 |
| BraA10g007420.3C | 4269820 | 4271213 | Ypt/Rab-GAP domain of gyp1p superfamily protein | 0.88 |
| BraA10g007430.3C | 4274091 | 4275250 | Involved in cell wall modifications resulting in resistance to the biotroph Hpa | 4.00E-30 |
| BraA10g007440.3C | 4285610 | 4286698 | The catalytic domain has the molecular function of riboflavin synthase | 0.17 |
| BraA10g007450.3C | 4295681 | 4299096 | MAP kinase | 0.44 |
| BraA10g007460.3C | 4302677 | 4303737 | YbaK/aminoacyl-tRNA synthetase-associated domain-containing protein | 0 |
| BraA10g007470.3C | 4326621 | 4327062 | NEP-interacting protein, putative (DUF239) | 1.4 |
| BraA10g007480.3C | 4327224 | 4331035 | Inner membrane OXA1-like protein | 0 |
| BraA10g007490.3C | 4347251 | 4348986 | Mitochondrial transcription termination factor family protein | 0.72 |
| BraA10g007500.3C | 4359690 | 4361252 | Encodes MCM2 (MINICHROMOSOME MAINTENANCE 2) | 0 |
| BraA10g007510.3C | 4365211 | 4365804 | Chaperone DnaJ-domain superfamily protein | 5.00E-80 |
| BraA10g007520.3C | 4372585 | 4374124 | Encodes one of two ubiquitin-conjugating enzymes belonging to the E2-C gene family (the other being UBC19) | 0.63 |
| BraA10g007530.3C | 4374893 | 4376044 | Transmembrane protein | 0.95 |
| BraA10g007540.3C | 4846779 | 4847021 | Member of MYB3R- and R2R3- type MYB- encoding genes | 0.015 |
| BraA10g007550.3C | 4851145 | 4852287 | ChaC-like family protein | 0 |
| BraA10g007560.3C | 4852717 | 4854984 | Translation initiation factor | 0 |
| BraA10g007570.3C | 4860497 | 4861888 | Elongation factor | 0 |
| BraA10g007580.3C | 4886648 | 4886938 | BTB/POZ protein that forms a complex with CUL3a | 0.064 |
| BraA10g007590.3C | 4891942 | 4892870 | Encodes a plasma membrane-localized ser/thr protein kinase | 0.057 |
| BraA10g007600.3C | 4913670 | 4913900 | Encodes a cytosolic prolyl-tRNA synthetase. | 0.61 |
| BraA10g007610.3C | 4922001 | 4922791 | Hypothetical protein | 0.13 |
| BraA10g007620.3C | 4950599 | 4952057 | Adenine nucleotide alpha hydrolases-like superfamily protein | 0 |
| BraA10g007630.3C | 4953125 | 4953328 | Hypothetical protein | 0.012 |
| BraA10g007640.3C | 4954814 | 4955699 | DNA-binding bromodomain-containing protein, interacts with core SWI/SNF complex components. | 0.6 |
| BraA10g007650.3C | 4977072 | 4984509 | Member of a family of proteins related to PUP1, a purine transporter | 0 |
| BraA10g007660.3C | 4988559 | 4991387 | DEAD box RNA helicase (PRH75) | 1.8 |
| BraA10g007670.3C | 4992190 | 4992969 | Encodes a member of the DREB subfamily A-2 of ERF/AP2 transcription factor family (DREB2B) | 0.5 |
| BraA10g007680.3C | 4994089 | 4994978 | RNA binding protein with nuclease activity essential for stress response | 0.2 |
| BraA10g007690.3C | 4995379 | 4997916 | Encodes an ABA- and drought-induced RING-DUF1117 gene | 0.086 |
| BraA10g007700.3C | 5007275 | 5007883 | Phosphofructokinase 7 | 4.00E-50 |
| BraA10g007710.3C | 5014414 | 5015760 | Encodes a subunit of chloroplasts chaperonins | 0.001 |
| BraA10g007720.3C | 5055134 | 5055664 | Encodes a subunit of chloroplasts chaperonins | 3.00E-38 |
| BraA10g007730.3C | 5080812 | 5081771 | Hypothetical protein | 2.00E-108 |
| BraA10g007740.3C | 5093486 | 5094133 | Encodes a defensin-like (DEFL) family protein | 0.15 |
| BraA10g007750.3C | 5097819 | 5099966 | Member of a large family of putative ligands homologous to Clavata3 | 0.57 |
| BraA10g007760.3C | 5106133 | 5107427 | Encoding PSII-S (CP22) | 0 |
| BraA10g007770.3C | 5140753 | 5143009 | Encodes chlorophyllide a oxygenase | 0 |
| BraA10g007780.3C | 5149681 | 5152918 | Ribosomal protein S5/Elongation factor G/III/V family protein | 0.17 |
| BraA10g007790.3C | 5158969 | 5159787 | Calmodulin-binding protein | 2.3 |
| BraA10g007800.3C | 5162983 | 5172856 | Encodes a protein similar to IAA amino acid conjugate hydrolase. | 0 |
| BraA10g007810.3C | 5174254 | 5176522 | Aldolase superfamily protein; | 0 |
| BraA10g007820.3C | 5222246 | 5223516 | Encodes an aldehyde dehydrogenase induced by ABA and dehydration | 0 |
| BraA10g007830.3C | 5224859 | 5226081 | HSP40/DnaJ peptide-binding protein | 0 |
| BraA10g007840.3C | 5244800 | 5247193 | Eukaryotic aspartyl protease family protein | 0 |
| BraA10g007850.3C | 5280797 | 5283638 | Amino acid permease 5 | 0 |
| BraA10g007860.3C | 5291814 | 5292879 | STAY-GREEN-like protein; | 0 |
| BraA10g007870.3C | 5305839 | 5307707 | Tetratricopeptide repeat (TPR)-like superfamily protein | 0 |
| BraA10g007880.3C | 5314329 | 5315913 | P-loop containing nucleoside triphosphate hydrolases superfamily protein | 0 |
| BraA10g007890.3C | 5316505 | 5320221 | Protein phosphatase 2C family protein | 0 |
| BraA10g007900.3C | 5320742 | 5323830 | Ras-related small GTPase | 0 |
| BraA10g007910.3C | 5329885 | 5331269 | Sequence-specific DNA binding transcription factor | 0 |
| BraA10g007920.3C | 5331912 | 5335160 | Encodes a transcriptional co-regulator of AGAMOUS | 0 |
| BraA10g007930.3C | 5339633 | 5340032 | One of 5 PO76/PDS5 cohesion cofactor orthologs of Arabidopsis. | 0.21 |
| BraA10g007940.3C | 5360265 | 5360974 | Member of plant specific copper transport protein family | 0.3 |
| BraA10g007950.3C | 5384076 | 5384264 | Encodes a SWI2/SNF2 chromatin remodeling protein belonging to the ISWI family | 0.011 |
| BraA10g007960.3C | 5399649 | 5399837 | Encodes a SWI2/SNF2 chromatin remodeling protein belonging to the ISWI family | 0.011 |
| BraA10g007970.3C | 5400415 | 5400756 | Tracheary element differentiation-related 6 | 4.00E-75 |
| BraA10g007980.3C | 5409933 | 5410340 | Protein kinase family protein | 3.9 |
| BraA10g007990.3C | 5410726 | 5412456 | Encodes Lil3:1 (light-harvesting-like) protein | 4.5 |
| BraA10g008000.3C | 5414631 | 5416361 | Pentatricopeptide repeat (PPR) superfamily protein | 0 |
| BraA10g008010.3C | 5426944 | 5429772 | Ubiquitin interaction motif-containing protein | 0 |
| BraA10g008020.3C | 5434466 | 5434654 | Zinc ion binding protein | 0.04 |
| BraA10g008030.3C | 5435798 | 5438651 | Encodes a UDP-glucose:sterol-glucosyltransferase | 0 |
| BraA10g008040.3C | 5469717 | 5470142 | ARM repeat superfamily protein | 1.2 |
| BraA10g008050.3C | 5499276 | 5500028 | Zinc knuckle (CCHC-type) family protein | 1.00E-10 |
| BraA10g008060.3C | 5500430 | 5500603 | Basic helix-loop-helix (bHLH) DNA-binding superfamily protein | 0.01 |
| BraA10g008070.3C | 5501103 | 5501633 | DNAse I-like superfamily protein | 3.00E-15 |
| BraA10g008080.3C | 5540161 | 5541314 | RING/U-box superfamily protein | 0.019 |
| BraA10g008090.3C | 5612282 | 5615218 | SSI is a plastidial enzyme and crucial for the synthesis of normal amylopectin in the leaves of Arabidopsis | 7.00E-11 |
| BraA10g008100.3C | 5615357 | 5617676 | Encodes a SKD1 (Suppressor of K+ Transport Growth Defect1) homolog | 0.033 |
| BraA10g008110.3C | 5618736 | 5619311 | Kinase associated protein phosphatase | 5.9 |
| BraA10g008120.3C | 5619799 | 5621869 | PWWP domain protein involved in regulation of FLC and flowering time | 0.8 |
| BraA10g008130.3C | 5623697 | 5624242 | Encodes a homolog of the S. cerevisiae Nop2 | 0.036 |
| BraA10g008140.3C | 5624479 | 5624905 | Low-density receptor-like protein | 0.73 |
| BraA10g008150.3C | 5625432 | 5626367 | Encodes a MADS box transcription factor | 0.17 |
| BraA10g008160.3C | 5626991 | 5627629 | A member of ARF GTPase family | 0.52 |
| BraA10g008170.3C | 5628251 | 5628775 | Encodes a SU(VAR)3-9 homolog, a SET domain protein | 0.12 |
| BraA10g008180.3C | 5629018 | 5629713 | Leucine-rich repeat protein kinase family protein | 0.004 |
| BraA10g008190.3C | 5629729 | 5634698 | Transmembrane protein | 0 |
| BraA10g008200.3C | 5702597 | 5703077 | Encodes an enzyme likely to act in tetrahydrofolate biosynthesis in vivo | 0.57 |
| BraA10g008210.3C | 5703485 | 5705770 | Encodes a hybrid proline-rich protein | 0.86 |
| BraA10g008220.3C | 5707185 | 5707963 | A member of the NPY gene family | 2.3 |
| BraA10g008230.3C | 5709346 | 5712630 | Tetratricopeptide repeat (TPR)-like superfamily protein | 1.3 |
| BraA10g008240.3C | 5759514 | 5762785 | E encodes receptorlike kinase (RLK) | 0 |
| BraA10g008250.3C | 5769252 | 5771766 | Encodes a membrane-bound endo-1,4-beta-D-glucanase, involved in cellulose biosynthesis | 0 |
| BraA10g008260.3C | 5796298 | 5799807 | Encodes a plasma membrane-located ferric chelate reductase | 0 |
| BraA10g008270.3C | 5899006 | 5899200 | Member of CYP706A | 0.14 |
| BraA10g008280.3C | 5899277 | 5899564 | Encodes a putative transcription factor | 0.77 |
| BraA10g008290.3C | 5903859 | 5910043 | Leucine rich receptor kinase | 0 |
| BraA10g008300.3C | 5910789 | 5914032 | Encodes a member of the exocyst complex gene family | 0 |
| BraA10g008310.3C | 5914659 | 5915926 | Encodes a cytosolic prolyl-tRNA synthetase. | 6.00E-43 |
| BraA10g008320.3C | 5915028 | 5915926 | Encodes a cytosolic prolyl-tRNA synthetase. | 1.00E-44 |
| BraA10g008330.3C | 5915028 | 5915926 | Encodes a cytosolic prolyl-tRNA synthetase. | 1.00E-44 |
| BraA10g008340.3C | 5930353 | 5934596 | Encodes a spindle assembly checkpoint protein MAD1 | 0 |
| BraA10g008350.3C | 5936317 | 5936880 | DNAJ heat shock N-terminal domain-containing protein | 1.6 |
| BraA10g008360.3C | 5937051 | 5937296 | Hypothetical protein (DUF3133) | 0 |
| BraA10g008370.3C | 5938149 | 5939282 | Encodes a protein containing the NFU domain and functions as a molecular scaffold for iron-sulfur cluster assembly and delivery | 0 |
| BraA10g008380.3C | 5950004 | 5950156 | Encodes a member of the cationic amino acid transporter (CAT) subfamily of amino acid polyamine choline transporters | 1.3 |
| BraA10g008390.3C | 5955163 | 5957545 | Encodes a nuclear localised protein MSA1 (MORE SULPHUR ACCUMULATION1) | 2.00E-44 |
| BraA10g008400.3C | 5959230 | 5960060 | HAD-superfamily hydrolase, subfamily IG, 5-nucleotidase | 1.00E-16 |
| BraA10g008410.3C | 5992467 | 5996066 | Alpha/beta-Hydrolases superfamily protein | 0 |
| BraA10g008420.3C | 6009302 | 6012305 | Xanthine/uracil permease family protein | 0 |
| BraA10g008430.3C | 6021309 | 6021608 | Encodes member of importin/exportin family | 0.42 |
| BraA10g008440.3C | 6022597 | 6024195 | Vacuolar sorting protein 39 | 0.32 |
| BraA10g008450.3C | 6025543 | 6025956 | Encodes a RING domain E3 ligase | 0.33 |
| BraA10g008460.3C | 6026006 | 6026260 | Encodes a cytosolic protein | 2.4 |
| BraA10g008470.3C | 6026912 | 6027609 | Plant invertase/pectin methylesterase inhibitor superfamily protein | 1.00E-148 |
| BraA10g008480.3C | 6042162 | 6043187 | Encodes a putative thioredoxin DCC1 involved in determining shoot regeneration capacity | 1.00E-160 |
| BraA10g008490.3C | 6074451 | 6075825 | Pentatricopeptide repeat (PPR) superfamily protein | 2 |
| BraA10g008500.3C | 6100208 | 6104811 | NAD(P)-binding Rossmann-fold superfamily protein; | 0 |
| BraA10g008510.3C | 6151752 | 6159904 | LOTR1 protein has an unknown function | 0 |
| BraA10g008520.3C | 6162939 | 6163305 | Leucine-rich repeat (LRR) family protein; | 2.5 |
| BraA10g008530.3C | 6273544 | 6273852 | 6-phosphogluconate dehydrogenase family protein; | 0.84 |
| BraA10g008540.3C | 6274141 | 6275553 | Involved in growth adaptation upon exposure to metal ions | 0.061 |
| BraA10g008550.3C | 6295689 | 6299791 | C2 calcium/lipid-binding and GRAM domain containing protein | 0 |
| BraA10g008560.3C | 6305147 | 6306158 | Encodes a high-affinity nitrate transporter | 7.00E-145 |
| BraA10g008570.3C | 6313015 | 6315802 | Encodes an Fe-S binding protein with quinolinate synthase (QS) activity and cysteine desulfurase activator activity | 0 |
| BraA10g008580.3C | 6316226 | 6318038 | Autophagy-related (ATG) gene | 0 |
| BraA10g008590.3C | 6334062 | 6334445 | Zinc finger (C2H2 type) family protein | 0.3 |
| BraA10g008600.3C | 6335939 | 6337534 | Encodes a homolog of the adenine-guanine-hypoxanthine transporter AzgA of Aspergillus nidulans | 0 |
| BraA10g008610.3C | 6351030 | 6352150 | DNA/RNA polymerases superfamily protein;( | 1.00E-7 |
| BraA10g008620.3C | 6366927 | 6369459 | Ribosomal RNA-processing protein | 0.076 |
| BraA10g008630.3C | 6403350 | 6404326 | Encoded by the Myb-like transcription factor MYB38, regulates axillary meristem formation. | 0.49 |
| BraA10g008640.3C | 6407571 | 6410373 | Protein kinase superfamily protein | 0 |
| BraA10g008650.3C | 6437804 | 6438439 | F-box/LRR protein | 0.45 |
| BraA10g008660.3C | 6448899 | 6449123 | Hypothetical protein | 3.00E-67 |
| BraA10g008670.3C | 6458335 | 6458879 | CORD2 is a member of a novel and plant specific family of microtubule associated proteins | 1.00E-68 |
| BraA10g008680.3C | 6460636 | 6461124 | Serine/Threonine-kinase, putative | 4.00E-95 |
| BraA10g008690.3C | 6461992 | 6464786 | Histone superfamily protein | 0.009 |
| BraA10g008700.3C | 6474240 | 6474566 | TCP-1/cpn60 chaperonin family protein | 3.1 |
| BraA10g008710.3C | 6476459 | 6481154 | Encodes a member of the DREB subfamily A-5 of ERF/AP2 transcription factor family (RAP2.10) | 0.24 |
| BraA10g008720.3C | 6504113 | 6504436 | Methyltransferase family protein | 0.88 |
| BraA10g008730.3C | 6540130 | 6540882 | Zinc knuckle (CCHC-type) family protein | 4.00E-09 |
| BraA10g008740.3C | 6541285 | 6541458 | Basic helix-loop-helix (bHLH) DNA-binding superfamily protein | 0.036 |
| BraA10g008750.3C | 6541957 | 6542487 | DNAse I-like superfamily protein | 4.00E-14 |
| BraA10g008760.3C | 6553519 | 6554076 | Disease resistance protein (CC-NBS-LRR class) family | 0.45 |
| BraA10g008770.3C | 6554148 | 6554743 | Member of Nramp2 family | 0.1 |
| BraA10g008780.3C | 6554752 | 6554913 | Ferritin/ribonucleotide reductase-like family protein | 1.4 |
| BraA10g008790.3C | 6566749 | 6572183 | Encodes MRL1, a conserved pentatricopeptide repeat protein | 0.25 |
| BraA10g008800.3C | 6730959 | 6733699 | PIF1 helicase | 0.067 |
| BraA10g008810.3C | 6734334 | 6734847 | Ribonuclease H-like superfamily protein | 0.21 |
| BraA10g008820.3C | 6753247 | 6755117 | Encodes the transcriptional regulator MED12 | 0.8 |
| BraA10g008830.3C | 6762646 | 6764727 | A member of EXO70 gene family, putative exocyst subunits | 0 |
| BraA10g008840.3C | 6776433 | 6778626 | Pentatricopeptide repeat (PPR-like) superfamily protein | 0 |
| BraA10g008850.3C | 6782852 | 6784852 | Transmembrane protein | 0.005 |
| BraA10g008860.3C | 6788372 | 6791990 | Protein phosphatase 2C family protein | 1.00E-22 |
| BraA10g008870.3C | 6793155 | 6793903 | Copper transport protein family | 3.2 |
| BraA10g008880.3C | 6795007 | 6795213 | Transmembrane/coiled-coil protein | 0.044 |
| BraA10g008890.3C | 6797932 | 6800194 | Transmembrane/coiled-coil protein | 0.27 |
| BraA10g008900.3C | 6806627 | 6806830 | Transmembrane/coiled-coil protein | 6.00E-64 |
| BraA10g008910.3C | 6809424 | 6810032 | Member of a family of F-Box proteins | 3.00E-04 |
| BraA10g008920.3C | 6815460 | 6815717 | NAC domain protein | 0.003 |
| BraA10g008930.3C | 6837265 | 6837456 | Encodes an asparaginase that catalyzes the degradation of L-asparagine to L-aspartic acid and ammonia | 0.14 |
| BraA10g008940.3C | 6837287 | 6838093 | LOB domain-containing protein 10 | 1.9 |
| BraA10g008950.3C | 6837287 | 6838690 | RNA recognition motif (RRM)-containing protein | 0.31 |
| BraA10g008960.3C | 6838800 | 6839915 | Ncodes CDK (cyclin-dependent kinase) inhibitor (CKI) | 0.019 |
| BraA10g008970.3C | 6839616 | 6839915 | Encodes an active Arabidopsis isopropylmalate synthase IPMS1 | 0.23 |
| BraA10g008980.3C | 6839616 | 6839915 | Encodes an active Arabidopsis isopropylmalate synthase IPMS1 | 0.23 |
| BraA10g008990.3C | 6839924 | 6840166 | P-loop containing nucleoside triphosphate hydrolases superfamily protein | 2.2 |
| BraA10g009000.3C | 6839924 | 6840166 | P-loop containing nucleoside triphosphate hydrolases superfamily protein | 2.2 |
| BraA10g009010.3C | 6839924 | 6840166 | P-loop containing nucleoside triphosphate hydrolases superfamily protein | 2.2 |
| BraA10g009020.3C | 6839924 | 6840166 | P-loop containing nucleoside triphosphate hydrolases superfamily protein | 2.2 |
| BraA10g009030.3C | 6843328 | 6846065 | Encodes a putative hydroxysteroid dehydrogenase (HSD) | 0 |
| BraA10g009040.3C | 6873185 | 6873891 | MORC4 is a member of a family of GHKL ATPases | 4.00E-55 |
| BraA10g009050.3C | 6901488 | 6902038 | MATH domain/coiled-coil protein | 0.09 |
| BraA10g009060.3C | 6931200 | 6933399 | Encodes a member of the SWEET sucrose efflux transporter family proteins | 0 |
| BraA10g009070.3C | 6954862 | 6955716 | ATPase E1-E2 type family protein | 0.02 |
| BraA10g009080.3C | 6975658 | 6976425 | Hypothetical protein | 0.001 |
| BraA10g009090.3C | 6977070 | 6977940 | Member of eIF3c - eukaryotic initiation factor 3c | 0.66 |
| BraA10g009100.3C | 6986046 | 6986255 | Lectin protein kinase family protein | 0.54 |
| BraA10g009110.3C | 6986505 | 6987002 | bZIP transcription factor family protein | 0.11 |
| BraA10g009120.3C | 6987102 | 6988421 | ARM repeat superfamily protein | 0.71 |
| BraA10g009130.3C | 6990929 | 6991228 | EEIG1/EHBP1 protein amino-terminal domain protein | 0.23 |
| BraA10g009140.3C | 6991459 | 6991647 | Hypothetical protein | 1.7 |
| BraA10g009150.3C | 6991722 | 6992522 | Encodes the Arabidopsis RAD50 homologue | 0.26 |
| BraA10g009160.3C | 7016414 | 7017027 | DNA polymerase delta small subunit; | 0.9 |
| BraA10g009170.3C | 7025324 | 7025788 | Required for repair of pyrimidine-pyrimidinone (6-4) dimers | 0.021 |
| BraA10g009180.3C | 7027929 | 7028428 | Encodes an ornithine delta-aminotransferase | 2.6 |
| BraA10g009190.3C | 7033485 | 7034218 | NAC domain containing protein 97 | 0 |
| BraA10g009200.3C | 7035376 | 7036860 | Nuclear polyadenylated RNA-binding protein | 0 |
| BraA10g009210.3C | 7037153 | 7037491 | Encodes FtsHi1 | 0.93 |
| BraA10g009220.3C | 7072744 | 7075807 | Transketolase family protein | 0 |
| BraA10g009230.3C | 7094881 | 7097367 | Protein kinase superfamily protein | 0 |
| BraA10g009240.3C | 7099530 | 7099820 | Receptor like protein 41 | 0.172 |
| BraA10g009250.3C | 7109374 | 7109676 | Transmembrane magnesium transporter | 0.0148 |
| BraA10g009260.3C | 7120797 | 7121295 | Encodes acyl-CoA-binding protein with ankyrin repeats | 6.00E-34 |
| BraA10g009270.3C | 7136934 | 7138339 | Alpha/beta-Hydrolases superfamily protein;(source:Araport11) | 0 |
| BraA10g009280.3C | 7138683 | 7144182 | Pyridine nucleotide-disulfide oxidoreductase family protein | 0 |
| BraA10g009290.3C | 7148857 | 7149978 | Encodes succinate dehydrogenase assembly factor 2 (SDHAF2) | 0 |
| BraA10g009300.3C | 7155127 | 7156921 | Encodes an APC isoform in Arabidopsis, a calcium-dependent mitochondrial ATP-Mg/Pi transporter | 0 |
| BraA10g009310.3C | 7162568 | 7164612 | Encodes a major determinant of natural variation in Arabidopsis flowering time | 4.00E-134 |
| BraA10g009320.3C | 7164775 | 7165688 | Fe superoxide dismutase | 6.00E-111 |
| BraA10g009330.3C | 7175629 | 7177125 | Encodes a homolog of the protein PABN1, a polyadenylation factor subunit. | 0 |
| BraA10g009340.3C | 7181614 | 7183628 | Encodes an ABA responsive C2H2-type zinc finger transcription factor | 0 |
| BraA10g009350.3C | 7187983 | 7190114 | Mitochondrial import inner membrane translocase subunit | 0 |
| BraA10g009360.3C | 7190623 | 7192769 | U6 snRNA phosphodiesterase-like protein | 0 |
| BraA10g009370.3C | 7207771 | 7208920 | Basic helix-loop-helix (bHLH) DNA-binding superfamily protein | 2.00E-55 |
| BraA10g009380.3C | 7215080 | 7215556 | Encodes a subtilisin-like serine protease essential for mucilage release from seed coats | 0.21 |
| BraA10g009390.3C | 7217328 | 7217741 | Encodes a member of the RLCK VII-4 subfamily of receptor-like cytoplasmic kinases | 1.00E-15 |
| BraA10g009400.3C | 7228107 | 7228298 | tRNA/rRNA methyltransferase (SpoU) family protein | 2.00E-30 |
| BraA10g009410.3C | 7235064 | 7237130 | Alpha/beta-Hydrolases superfamily protein | 0 |
| BraA10g009420.3C | 7238230 | 7238883 | Encodes a member of the ERF (ethylene response factor) subfamily B-3 of ERF/AP2 transcription factor family | 0 |
| BraA10g009430.3C | 7242268 | 7242853 | Encodes oleosin3, involved in seed lipid accumulation. | 1.00E-121 |
| BraA10g009440.3C | 7246986 | 7248099 | Ubiquinol-cytochrome C chaperone family protein | 0 |
| BraA10g009450.3C | 7255320 | 7257839 | Encodes a protein that is highly methylated in a WT DML background. | 0.28 |
| BraA10g009460.3C | 7260699 | 7260911 | PLAT/LH2 domain-containing lipoxygenase family protein | 0.4 |
| BraA10g009470.3C | 7262807 | 7263229 | Glycine-rich protein | 0.006 |
| BraA10g009480.3C | 7264509 | 7264889 | Involved in the second step of nitrate assimilation | 2.00E-47 |
| BraA10g009490.3C | 7274730 | 7275584 | Transmembrane protein | 4.00E-08 |
| BraA10g009500.3C | 7274730 | 7275584 | Transmembrane protein | 4.00E-08 |
| BraA10g009510.3C | 7284004 | 7286662 | 6,7-dimethyl-8-ribityllumazine synthase | 0.004 |
| BraA10g009520.3C | 7286671 | 7286913 | Encodes a vacuole localized protein of the ABC transporter White-Brown Complex (WBC) family | 7.8 |
| BraA10g009530.3C | 7302773 | 7303126 | Sucrase/ferredoxin-like family protein | 0.76 |
| BraA10g009540.3C | 7318965 | 7321024 | Regulates the meristem response to light signals and the maintenance of inflorescence meristem identity | 0.21 |
| BraA10g009550.3C | 7318986 | 7319564 | P-loop containing nucleoside triphosphate hydrolases superfamily protein | 1.6 |
| BraA10g009560.3C | 7415559 | 7416094 | Telomerase activating protein Est1 | 0.1 |
| BraA10g009570.3C | 7419974 | 7420303 | Encodes a cytosolic ascorbate peroxidase APX1 | 3.1 |
| BraA10g009580.3C | 7420791 | 7421213 | Regulates DNA replication via interaction with BICE1 and MCM7 | 0.028 |
| BraA10g009590.3C | 7527310 | 7529661 | Galactose mutarotase-like superfamily protein | 0.21 |
| BraA10g009600.3C | 7565658 | 7565918 | Encodes FERRITIN 3 | 0.2 |
| BraA10g009610.3C | 7579675 | 7582653 | Zinc knuckle (CCHC-type) family protein | 8.00E-11 |
| BraA10g009620.3C | 7646430 | 7652935 | DNAJ heat shock N-terminal domain-containing protein | 0.15 |
| BraA10g009630.3C | 7654593 | 7654847 | Structural maintenance of chromosomes domain protein | 0.19 |
| BraA10g009640.3C | 7666615 | 7667742 | AGAMOUS-like 93 | 0.14 |
| BraA10g009650.3C | 7669144 | 7677000 | Chitinase family protein | 1.00E-105 |
| BraA10g009660.3C | 7709545 | 7709985 | Member of Putative ligand-gated ion channel subunit family | 0.35 |
| BraA10g009670.3C | 7725759 | 7726980 | Encodes a protein with similarity to histone deacetylases | 4.00E-38 |
| BraA10g009680.3C | 7734152 | 7736083 | O-glucosyltransferase rumi-like protein (DUF821) | 0.21 |
| BraA10g009690.3C | 7740225 | 7743490 | Catalytics | 0.55 |
| BraA10g009700.3C | 7745719 | 7755217 | Encodes a member of the DREB subfamily A-6 of ERF/AP2 transcription factor family | 0.54 |
| BraA10g009710.3C | 7768187 | 7768687 | Regulates the meristem response to light signals and the maintenance of inflorescence meristem identity | 0.4 |
| BraA10g009720.3C | 7773955 | 7775470 | Encodes a plant protein kinase similar to the calcium/calmodulin-dependent protein kinase subfamily and the SNF1 kinase subfamily (SnRK2) | 0.002 |
| BraA10g009730.3C | 7786359 | 7788123 | One of the type IIB calcium pump isoforms | 0.12 |
| BraA10g009740.3C | 7815861 | 7821350 | Glycine-rich protein | 0.043 |
| BraA10g009750.3C | 7824582 | 7826438 | Glycine-rich protein family | 2.00E-33 |
| BraA10g009760.3C | 7884442 | 7884726 | Encodes a phytochrome photoreceptor with a function similar to that of phyB | 0.77 |
| BraA10g009770.3C | 7886614 | 7886946 | Elongation factor family protein | 0.26 |
| BraA10g009780.3C | 7914973 | 7915314 | Tetratricopeptide repeat (TPR)-like superfamily protein | 0.27 |
| BraA10g009790.3C | 8013156 | 8013671 | A20/AN1-like zinc finger family protein | 1.00E-134 |
| BraA10g009800.3C | 8028688 | 8029301 | Cysteine/Histidine-rich C1 domain family protein | 7.00E-04 |
| BraA10g009810.3C | 8074977 | 8077483 | ARM repeat superfamily protein | 1.00E-38 |
| BraA10g009820.3C | 8084393 | 8085438 | Polycomb group protein with zinc finger domain involved in negative regulation of reproductive development | 1.00E-82 |
| BraA10g009830.3C | 8095135 | 8099924 | Polycomb group protein with zinc finger domain involved in negative regulation of reproductive development | 0 |
| BraA10g009840.3C | 8105038 | 8106292 | SNF7 family protein | 2.00E-51 |
| BraA10g009850.3C | 8106783 | 8107653 | HAD superfamily, subfamily IIIB acid phosphatase | 0 |
| BraA10g009860.3C | 8113384 | 8116476 | Plant U-box type E3 ubiquitin ligase (PUB) | 0 |
| BraA10g009870.3C | 8129325 | 8134845 | Plant U-box type E3 ubiquitin ligase (PUB) | 0 |
| BraA10g009880.3C | 8139415 | 8139693 | Ppredicted to encode a protein that functions as a Golgi apparatus structural component known as a golgin in mammals and yeast | 0.21 |
| BraA10g009890.3C | 8141177 | 8141656 | Zinc ion binding protein | 3.00E-26 |
| BraA10g009900.3C | 8142116 | 8142727 | Hexokinase | 0.14 |
| BraA10g009910.3C | 8143660 | 8145626 | Plant U-box type E3 ubiquitin ligase (PUB). | 3.00E-58 |

Table S6 Gene information of *BraA10g007770.3C* (*BrCAO*)

| Type | Start | End | Direction | Gene ID | Length |
| --- | --- | --- | --- | --- | --- |
| gene | 5140753 | 5143009 | + | ID=BraA10g007770.3C | 2257 |
| mRNA | 5140753 | 5143009 | + | ID=BraA10g007770.3C | 2257 |
| CDS | 5140753 | 5140839 | + | Parent=BraA10g007770.3C | 87 |
| CDS | 5140933 | 5141169 | + | Parent=BraA10g007770.3C | 237 |
| CDS | 5141241 | 5141345 | + | Parent=BraA10g007770.3C | 105 |
| CDS | 5141422 | 5141691 | + | Parent=BraA10g007770.3C | 270 |
| CDS | 5141781 | 5141928 | + | Parent=BraA10g007770.3C | 148 |
| CDS | 5142007 | 5142191 | + | Parent=BraA10g007770.3C | 185 |
| CDS | 5142265 | 5142377 | + | Parent=BraA10g007770.3C | 113 |
| CDS | 5142462 | 5142756 | + | Parent=BraA10g007770.3C | 295 |
| CDS | 5142845 | 5143009 | + | Parent=BraA10g007770.3C | 165 |

Table S7 Primer sequences used for clone sequencing.

| Primer | Forward sequence (5’-3’) | Reverse sequence (5’-3’) |
| --- | --- | --- |
| FL-BrCAO-1 | TAATCTCTTCGTCTATTGTCTTCCT | AACATTTTTATACATACCATCGTGTC |
| FL-BrCAO-2 | TTAGACAGGGAAAAGACAAACATT | ACTCAGACATGCAGAGAGAACG |
| CDS-BrCAO-1 | ACGCCGCCGTGTTTACT | TTCCCAGGTTTTGAGATTCC |
| CDS-BrCAO-2 | ACAAGACGGTATGGTCTGGGT | TTAGCCTGAGAAAGGTAATTTATCA |

Table S8 Primer sequences used for qRT-PCR.

| Primer | Forward sequence (5’-3’) | Reverse sequence (5’-3’) |
| --- | --- | --- |
| qRT-BrCAO | TTTTTTTTAATTGGTGCTCGCT | AAGGTCTGTCTGATTCTGTCGC |
| ACTIN | ATCTACGAGGGTTATGCT | CCACTGAGGACGATGTTT |
